# A new species and a key to the species of *Corticarina* from Guatemala (Coleoptera, Latridiidae)

**DOI:** 10.3897/zookeys.786.26553

**Published:** 2018-09-25

**Authors:** Jose Carlos Otero, José Manuel Pereira

**Affiliations:** 1 University of Santiago de Compostela, Department of Zoology, Genetics and Physical Anthropology, Santiago de Compostela 15782, Spain University of Santiago de Compostela Santiago de Compostela Spain

**Keywords:** *Corticarinaoscargloriorum* sp. n., Guatemala, new species, taxonomic key, taxonomy

## Abstract

A new species of *Corticarina* Reitter, 1881 (Coleoptera: Latridiidae), *Corticarinaoscargloriorum***sp. n.** from Guatemala is described and illustrated. The differential diagnosis is established in relation to a group of other species of the genus.

## Introduction

Latridiidae is a moderately large family with approximately 500 species which is represented in all major biogeographic regions. The genus *Corticarina* is distributed in the Holarctic, Neotropical, Afrotropic and Oriental regions (Rücker 2009). In Central America, the fauna of Corticariinae is presumably extraordinarily rich, however, little known despite the attention that some authors (Johnson 1978, 1981, 1997; Reike 2015; Rücker 1981a, 1981b, 1987; Sharp 1902) have paid to some genera of this family (*Melanophthalma* Motschulsky, *Corticarina* Reitter, *Cortinicara* Johnson, etc.). Twenty species of *Corticarina* are known from Central America. Four of them, considered in this article, are distributed exclusively in Guatemala (Rücker 2009). As a result of that work, we have had the opportunity to study additional material collected in Central America (Zoological Museum, University of Lund, Sweden). Presumably most species considered in this article are endemic to Guatemala. The present paper presents part of the material collected at Sierra Madre Oriental in Guatemala.

## Materials and methods

The terminology and the measurements of the new species follow Otero (1997) and Otero and López (2016). Structures were measured under a Leica M205C- stereomicroscope equipped with an Application Suite analysis system.

Abbreviations:

**L** length;

**WL** width/length ratio;

**E** eccentricity of the eyes (width/half of the length).

The width is measured across the widest part of a line joining the anterior and posterior limit of the eye. Length is the maximum length of the eye. **L** is used for length in dorsal view, **W** for width, and **Ø** for diameter.

Institutional abbreviations:

**USC**Universidad de Santiago de Compostela, Spain;

**ZML**Zoological Museum, University of Lund, Sweden.

## Taxonomy

### Gen. Corticarina

Taxon classificationAnimaliaColeopteraLatridiidae

Reitter, 1881

#### Type species.

*Corticarinatruncatella* Mannerheim, 1844

#### Diagnosis.

Head generally much narrower than the pronotum. Pronotum rather broad, usually strongly curved at the sides, with or without a circular post median impression; hind angles clearly toothed. Basal segment of the tarsi (especially the hind) strongly produced ventrally so that its apex almost reaches the apex of the second segment; second tarsomere reduced, arising dorsally from the basal segment nearly at the middle. Male: front tibia with a tooth situated at or a little in front of the middle; aedeagus strongly sclerotized, asymmetrical, the ostium ventral and covered with a plate which is usually strongly projecting apically.

### Corticarina oscargloriorum sp. n.

Taxon classificationAnimaliaColeopteraLatridiidae

http://zoobank.org/8A6EE954-93DB-49BB-9E47-B635F64AD1DE

Figures 1–4

#### Material examined.

“Holotype m*. **GUATEMALA.** Jalapa, Pino Dulce, Mataquescuintla, 14.5256528°N, 90.1453500°W, 28.X.2016, 2.400 m”. Holotype placed in Coll. J. C. Otero (USC).

#### Diagnosis.

Morphologically, *Corticarinaoscargloriorum* is very similar to other *Corticarina* in many external features, but can be distinguished by the configuration of the male genital apparatus and the features in the key below.

#### Description.

**Figures. 1–4.**
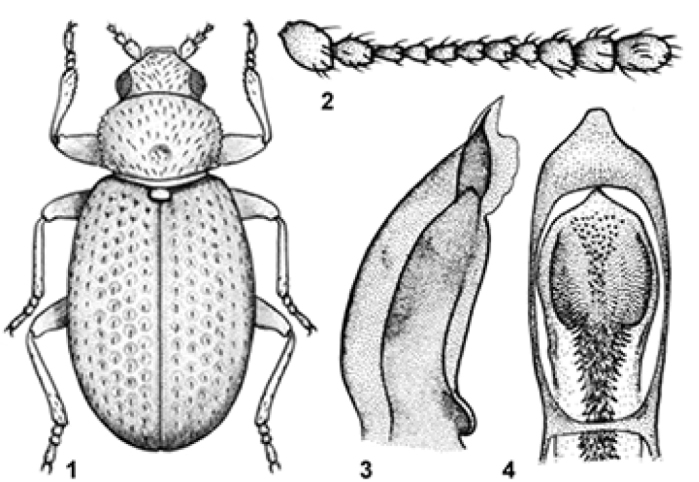
*Corticarinaoscargloriorum* sp. n., male, holotype: (**1**) habitus; (**2**) antenna; (**3–4**) aedeagus (lateral and ventral views).

Length: 1.1 mm. Body oval, convex and little bright (Figure 1). Yellowish grey-brown; antennae (except for the two first articles) and tibiae dark grey-brown; two first articles of antennae and legs yellowish grey-brown. Metathoracic wings fully developed.

*Head* (Figure 1) slightly transverse (WL = 1.8–1.9). Labrum arcuate at anterior margin. Eyes large (L = 0.073 mm) and little protruding (E = 0.5–0.6); eye facets as large as head punctures. Puncturation very fine (Ø = 0.006–0.007 mm) and sparse, barely distinct. Temples indistinct. Antennae (Figure 2) long (L = 0.398 mm). First antennomere spherical, almost as wide as long; second slightly shorter and half as thin as first; from fourth to seventh almost identical and half as short as second; eighth as wide as long; ninth sub-conical, longer than wide and 1.2 times as long as tenth; tenth 1.1 times longer than wide; eleventh twice as long.

*Pronotum* (Figure 1) moderately convex and little transverse (WL = 1.2–1.3); greatest width at anterior third. Lateral margins regularly rounded and denticulate. Postmedial circular depression present; lateral impressions somewhat distinct. Pubescence whitish, short (L = 0.019–0.020 mm) and recumbent. Punctation fine (Ø = 0.011–0.012 mm) and sparse.

*Elytra* 1.5 times as long as wide. Callosity humeralwell-marked and posteriorly prolonged until becoming slightly carenated in the humeral region. Pubescence short (L = 0.016–0.018 mm) and recumbent. Punctation fine (Ø = 0.014 mm) and sparse. Six abdominal sternites visible. Male pro-tibiae with a tooth on internal apical third.

*Aedeagus* (Figs 3, 4) sub-lanceolate with a small apical protuberance. Internal sac with numerous small spines.

#### Etymology.

This species is dedicated to Óscar Medinilla and Gloria de Dios, Guatemala.

#### Distribution.

Guatemala.

#### Biology.

It has been captured by stirring different types of plant formations.

### Key to the *Corticarina* species from Guatemala

**Table:** 

1	Tenth antennomere distinctly transverse; ninth squared. Spine of male pro-tibiae on the last quarter of the internal margin. Aedeagus (Fig. 5). L: 1.4–1.5 mm. Guatemala	***guatemalica* Johnson**
–	Tenth antennomere as long as wide or longer than wide	**2**
2	Ninth antennomere conical, wider than long; tenth as long as wide. Light brown; legs yellowish grey-brown; antennae pale on the base however darkening towards the apex. Spine of male pro-tibiae in the middle of the internal margin. Aedeagus (Fig. 7). L: 1.4–1.6 mm. Guatemala	***conjuncta* Johnson**
–	Ninth antennomere markedly longer than wide	**3**
3	Pale colour; head and pronotum slightly darker; antennae and legs entirely (except for the antennal club) yellowish grey-brown. Ninth antennomere conical and longer than wide; tenth longer than wide and slightly shorter than ninth. Spines of male pro-tibiae arranged on the middle of the internal margin. Aedeagus (Fig. 6). L: 1.4–1.6 mm. Guatemala	***impensa* Johnson**
–	Yellowish grey-brown; antennae (except for the two first articles) and tibiae dark greybrown; two first articles of the antennae and legs yellowish grey-brown. Ninth antennomere (Fig. 2) sub-conical, longer than wide; tenth 1.1 times longer than wide and slightly shorter than ninth. Spines of pro-tibiae on apical third. Aedeagus (Figs 3& 4). L: 1.1 mm. Guatemala	***oscargloriorum* sp. n.**

**Figures. 5–7.**
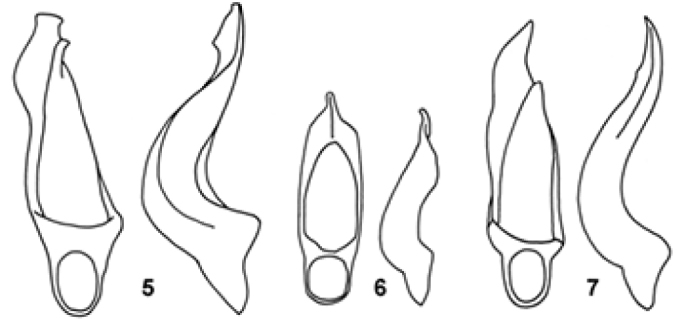
Adeadeagi of species of *Corticarina*, ventral and lateral views: (**5**) *Corticarinaguatemalica*; (**6**) *Corticarinaimpensa*; (**7**) *Corticarinaconjuncta*.

## Discussion

Four species for *Corticarina* have been recorded from the Guatemala to date, and the four species are known to the authors. Morphologically, *Corticarinaoscargloriorum* is very similar to other *Corticarina* which are remarkably uniform in appearance. Their male genitalia fortunately provide excellent diagnostic characters and are thus essential for identification.

## Supplementary Material

XML Treatment for Corticarina

XML Treatment for Corticarina oscargloriorum
